# Treatise on Surgery

**Published:** 1876-04

**Authors:** 


### Bibliographical.

A Treatise on Surgery—Its Principles and Practice. By T. Holmes, Cantab; Surgeon to St. George's Hospital. With 411 illustrations, chiefly by Dr. Westmacott. Philadelphia: Henry C. Lea, publisher. Book, pp. 960.

This work, just from the press, is divided into forty-four chapters, contains nearly 1000 pages, and is interspersed throughout with many illustrations. The 1st chapter is devoted to inflammation, process of union in soft parts, dressing wounds, etc.; 2d, to complications of wounds; 3d, poisoned wounds; 4th, hemorrhage; 5th, burns and scalds; 6th, fractures and dislocations; 7th to 15th, devoted to injuries; 16th, gunshot wounds; 17th, tumors; 18th, scrofula; 19th, hysteria; 20th, gonorrhœa and syphilis; 21st, ulcers, etc.; 22d, diseases of bone; 23d, diseases of joints; 24th, diseases of spine; 25th, 26th, club-foot; 27th, affections of nerves; 28th and 29th, diseases of arteries and veins; 30th, surgical diseases of head and face; 31st, surgical diseases of digestion tract; 32d, hernia; 33d to 37th, diseases of rectum, larynx, eye and ear, and urinary organs; 38th, calculus; 39th and 40th, diseases of male and female organs of generation; 41st, diseases of breast; 42d, diseases of thyroid body; 43d, diseases of skin; 44th, minor surgery.

It will be seen from the table of contents that the work is full and the subjects treated of practical importance. Sold by Henry C. Lea, Philadelphia.

